# Acute right upper extremity ischemia resulting from true aneurysmof right brachial artery: A case report

**DOI:** 10.34172/jcvtr.2020.54

**Published:** 2020-11-24

**Authors:** Niki Tadayon, Sina Zarrintan, Seyed Mohammad Reza Kalantar-Motamedi

**Affiliations:** ^1^Division of Vascular & Endovascular Surgery, Department of General & Vascular Surgery, Shohada-Tajrish Medical Center, Shahid Beheshti University of Medical Sciences, Tehran, Iran; ^2^Cardiovascular Research Center, Tabriz University of Medical Sciences, Tabriz, Iran

**Keywords:** Brachial Artery, True Aneurysm, Revascularization, Acute Limb Ischemia

## Abstract

We report a case of 66-year-old woman with true aneurysm of the right brachial artery. She presented with acute upper extremity ischemia. The hand was cold and parenthesized and distal pulses were absent. CT angiography (CTA) revealed a 20*25 mm true brachial artery aneurysm. The aneurysm was thrombosed without distal run-off. We excised the aneurysm and reestablished the arterial flow by a reverse saphenous interposition graft. The postoperative course was uneventful.

## Introduction

True aneurysm of brachial artery is a rare entity. Peripheral aneurysms of upper limb result from trauma, thoracic outlet syndrome or creation of arteriovenous fistulas (AVF) for dialysis. They also may be iatrogenic.^1,2^ However, true aneurysms of brachial artery are uncommon and may cause acute limb ischemia. They may be diagnosed by a precise physical examination as a pulsatile mass. Most of these aneurysms remain asymptomatic until a complication occurs. Delayed diagnosis may cause significant morbidity.^3^

Herein, we present a case of true aneurysm in right brachial artery which was complicated by distal embolization and acute limb ischemia. Management of acute ischemia and anatomical revision of the aneurysm is of potential clinical and surgical interest in the present case.

## Case Presentation

We report a rare case of true right brachial artery aneurysm. The patient was a 66-year-old woman who was referred to our center because of acute limb ischemia. The patient complained of coldness and pain of right hand and forearm since 12 hours. In physical examination, the hand and forearm were cold and paresthesia was present in her hand distal to the wrist. Flexion and extension of wrist and fingers were diminished. Distal radial and ulnar pulses were absent. A 2*2 cm mass was present at right antecubital region. Examination of other organs was normal.

**Figure 1:**
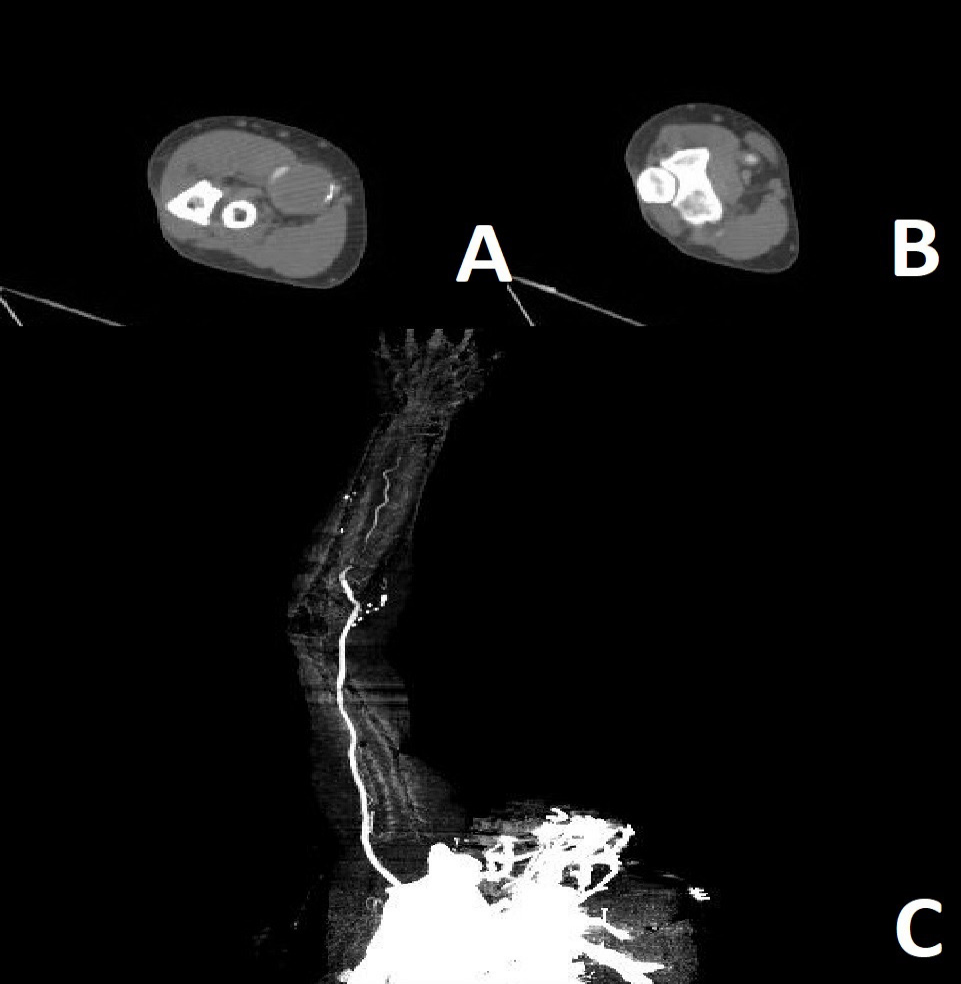
CT angiogram of the patient. True aneurysm of right brachial artery is seen with thrombosis inside the aneurysm sac (A), normal arterial flow is present in brachial artery proximal to the aneurysm (B), distal emboli to right radial and ulnar arteries, run-off is absent in these arteries (C)

**Figure 2:**
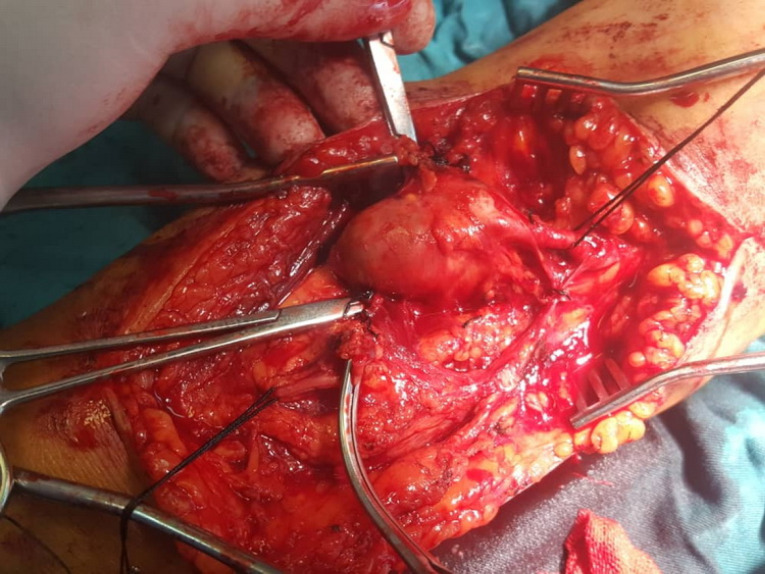
Intraoperative illustration of true aneurysm of right brachial artery

A CT angiography (CTA) was done. An aneurysm measuring 20*25 mm was present at distal part of brachial artery extending to its bifurcation (Figure 1). Normal arterial flow was present proximal to the aneurysm. The aneurysm was thrombosed and distal radial and ulnar arteries did not have contrast run-off. It was assumed that the aneurysm was complicated by distal embolization and the patient had acute limb ischemia. Thus, urgent surgical exploration and vascular reconstruction was planned. The patient was anesthetized and antecubital incision was done. Brachial artery was explored and proximal control was obtained. A true brachial artery aneurysm measuring 25*25*30 mm was present at distal part extending to the bifurcation (Figure 2). Radial and ulnar arteries were stenotic. The median nerve was also explored and was separated from the aneurysm. After heparin administration and proximal clamping, the aneurysm was incised and then resected. A 2-French Fogarty catheter was used to remove distal emboli. Radial artery was ligated. A reverse saphenous vein graft was interposed between brachial and ulnar arteries to perform arterial interposition. Proximal and distal anastomoses were performed by 6-0 and 7-0 polypropylene sutures respectively. Distal ulnar pulse was present after revascularization. The limb was warm two hours after the operation. Motor and sensory examinations were normal at following postoperative days. The patient had a normal right upper extremity examination at three-month and six-month follow-up visits.

## Discussion

Although true brachial artery aneurysms are rare, they can be complicated by distal embolization and may cause acute limb ischemia. Precise diagnosis of brachial artery aneurysms is necessary to prevent irreversible events.^1-3^ Resection of brachial artery aneurysms necessitates interposition graft for revascularization. Herein, we reported a case of true brachial artery aneurysm which was been complicated by acute limb ischemia because of acute distal emboli. Immediate and precise diagnosis and revascularization together with distal embolectomy is limb-saving and should be planned as soon as diagnosis is established.

Senarslan et al reported three cases of true brachial aneurysms which all had been complicated by distal embolization. They also used interposition grafts to restore the arterial flow.^1^ We also used reverse saphenous interposition graft to reestablish the arterial continuity. It seems that although endovascular intervention is a possible option, anatomical features and distal embolization would make it very difficult to exclude the aneurysm and restore the arterial flow. Thus, open surgical excision and revascularization is a safe and feasible technique in true brachial aneurysms. However, successful endovascular attempts to repair peripheral arterial aneurysms have been reported.^4^

Hudorović et al reported a case of true brachial artery aneurysm in a 77-year-old female which was discovered in CTA. The aneurysm was saccular and they used interposition graft to revascularize the aneurysmal part of the artery.^5^ It seems that excision of aneurysm of brachial artery necessitates interposition graft in most cases because tension between to normal ends of the artery precludes primary end-to-end anastomosis.

Surgical intervention for upper extremity arterial aneurysms should be performed without delay. Acute limb ischemia causes irreversible hand ischemia, acute compartment syndrome and gangrene. Then it results in amputation and considerable morbidity.^1,6,7^ However, true brachial artery aneurysms could present without ischemic signs. A painful pulsatile mass may be present. Even in patients without ischemic events, surgical intervention and interposition of an autologous vein is recommended.^8^ However, biological or prosthetic grafts may also be used in certain circumstances.^1^ The bifurcation of brachial artery was involved in our case. We ligated radial artery and placed the interposition graft between brachial and ulnar arteries. However, a bifurcated saphenous vein graft can also be used to revascularize both radial and ulnar arteries.^2^

Brachial artery aneurysms could also result from catheterization and drug abuse. In these circumstances, mycotic aneurysms will be present in the upper extremity arteries. The most common artery in upper extremity is brachial artery and the most common microorganism is gram positive bacteria.^9^ Our case was a true aneurysm of brachial artery. The patient did not have history of trauma or catheterization through brachial artery or surrounding vessels.

Brachial artery aneurysms cause thrombosis of the aneurysmal sac and distal embolization. This may cause ulceration and gangrenous lesions of the fingers.^1^ However, revascularization and distal embolectomy is recommended in patients with severe acute ischemia and ulcerations. Rest pain and ulceration usually respond dramatically to revascularization.^10^ However, recurrence of brachial artery aneurysm has also been reported.^11^ Our case also responded considerably to treatment and the postoperative course was uneventful. Thus, precise attention is necessary to prompt diagnosis and treatment of these relatively rare peripheral aneurysms.

Acute upper extremity ischemia can result from peripheral arterial aneurysms of the thoracic outlet, axillary region, brachial artery and even distal ulnar and radial arteries.^12-14^ Arterial aneurysms of upper extremity result from thoracic outlet syndrome (TOS), blunt or penetrating trauma, iatrogenic catheterization and placement of AVFs.^1,2^ In addition to acute limb ischemia, proximal aneurysms of upper extremity can be asymptomatic and present with a pulsatile mass. Open surgical repair and endovascular covered stent placement are two potential strategies to repair axillary and subclavian artery aneurysms.^15,16^ Blunt and penetrating trauma to brachial and axillary artery could potentially injure the vessel and cause pseudoaneurysms. Interposition with saphenous vein or prosthetic grafts are necessary to restore arterial flow in these circumstances.^17,18^ In addition, axillary artery aneurysms may be result of degenerative lesions often secondary to the chronic use of crutches.^12^ This condition can also cause aneurysm of the brachial artery.^19^ Crutch-induced brachial artery aneurysm is of potential clinical significance and may cause acute limb ischemia similar to other etiologies of upper extremity aneurysms.^20^ Although arterial TOS is one of the main causes of distal embolization and hand ischemia, in the present case the patient did not have TOS and the source of embolization was the true aneurysm of the brachial artery itself.

Arteriovenous fistulas for hemodialysis are common causes of brachial artery aneurysms. Following arteriovenous fistula creation, the increase of arterial flow can lead to aneurysmal degeneration, even after fistula ligation or renal transplant immunosuppression.^21^ Khalid et al reported three cases of brachial artery aneurysms following antecubital AVF ligation. Two of the cases presented with pulsatile mass while the other presented with limb ischemia.^22^ However, AVF-related brachial artery aneurysms can be found after AVF creation,^23^ and in kidney transplant patient who previously had AVF for hemodialysis.^24^ Thus, AVF related brachial artery aneurysms can be present while AVF is working, after AVF ligation and after transplantation. It may also result from trauma to AVF.^25^

## Conclusion

In conclusion, true aneurysms of the brachial artery are a relatively rare peripheral arterial aneurysm. They can be complicated by distal embolization and acute limb ischemia. This condition can cause limb ischemia, gangrene and considerable morbidity. Thus, prompt diagnosis and treatment of these aneurysms are necessary. Brachial artery aneurysms may be spontaneous. However, blunt and penetrating trauma, AVF creation, AVF ligation and chronic use of crutches can cause brachial artery aneurysms. We believe that open surgical repair preferably with autogenous vein graft is the treatment of choice for brachial artery aneurysms.

## Acknowledgements

The authors acknowledge Dr. Annis Shahnaee for her kind support during the preparation of this manuscript.

## Competing interest

The authors declare that they do not have any conflicts of interest.

## Ethical approval

The authors provided informed consent from the patient of the present case report. The informed consent was provided on the basis that the patient’s data are confidential and her name and other characteristics would remain blind during peer review, editorial process and publication. The signed consent is available upon request.

## Funding

None.
